# Does the unusual phenomenon of sustained force circumvent the speed–endurance trade-off in the jaw muscle of the southern alligator lizard (*Elgaria multicarinata*)?

**DOI:** 10.1242/jeb.247979

**Published:** 2025-01-27

**Authors:** Allyn Nguyen, Kyle Leong, Natalie C. Holt

**Affiliations:** Evolution, Ecology, and Organismal Biology Department, University of California, Riverside, Riverside, CA 92521, USA

**Keywords:** Muscle contraction, Contractile velocity, Twitch time, Contractile properties, Muscle fatigue, Feeding, Locomotion, Bite force

## Abstract

The jaw muscles of the southern alligator lizard, *Elgaria multicarinata*, are used in prolonged mate-holding behavior, and also to catch fast prey. In both males and females, these muscles exhibit an unusual type of high endurance known as sustained force in which contractile force does not return to baseline between subsequent contractions. This phenomenon is assumed to facilitate the prolonged mate-holding observed in this species. Skeletal muscle is often subject to a speed–endurance trade-off. Here, we determined the isometric twitch, tetanic and isotonic force–velocity properties of the jaw muscles at ∼24°C as metrics of contractile speed and compared these properties with a more typical thigh locomotory muscle to determine whether endurance by sustained force allows for circumvention of the speed–endurance trade-off. The specialized jaw muscle was generally slower than the more typical thigh muscle: time to peak twitch force, twitch 90% relaxation time (*P*<0.01), and tetanic 90% and 50% relaxation times (*P*<0.001) were significantly longer, and force–velocity properties were significantly slower (*P*<0.001) in the jaw than the thigh muscle. However, there seemed to be greater effects on relaxation rates and shortening velocity than on force rise times: there was no effect of muscle on time to peak, or 50% of tetanic force. Hence, the jaw muscle of the southern alligator lizard does not seem to circumvent the speed–endurance trade-off. However, the maintenance of force rise times despite slow relaxation, potentially enabled by the presence of hybrid fibers, may allow this muscle to meet the functional demand of prey capture.

## Teaser

**Summary:** The southern alligator lizard jaw muscles are used in mate-holding, and generally have slower contractile properties, yet retain somewhat faster activation kinetics that potentially allow them to meet conflicting demands.

## INTRODUCTION

Skeletal muscles produce the mechanical output required for organisms to interact with their environment, and thus are an important determinant of fitness (Husak et al., 2006; Lappin and Husak, 2005). This mechanical output is achieved through the calcium-mediated interactions between the contractile proteins actin, myosin (Gordon et al., 2000; Kuo and Ehrlich, 2015) and titin (Dutta et al., 2018; Nishikawa et al., 2019). However, despite this common mechanism of contraction, considerable variation in muscle and organismal performance is observed across muscles and species (Hoyle, 1967; Rome et al., 1988; Biewener, 1998; Wilson and Lichtwark, 2011; Mendoza et al., 2023). The long pectoralis muscle fibers of birds allows them to meet the substantial work demands of flight (Biewener, 1998), the slow red and fast white myotomal muscle of fish allows for both endurance and sprint swimming (Rome et al., 1988), and the sustained force produced by the amplexus muscle of frogs (Peters and Aulner, 2000; Navas and James, 2007) and the jaw muscles of the southern alligator lizard (*Elgaria multicarinata*) (Nguyen et al., 2020) is thought to facilitate prolonged mate-holding. However, this variation in muscle performance may be constrained by trade-offs, particularly the speed–endurance trade-off (Schmidt-Nielsen, 1984; Garland, 2014; Castro et al., 2022; Garland et al., 2022).

Much of the variation in skeletal muscle performance (Rome et al., 1988; William et al., 1997; Hyatt et al., 2010; Kohn et al., 2011; Spainhower et al., 2018), and the often-observed speed–endurance trade-off (Vanhooydonck et al., 2001; Bonine et al., 2005), is attributed to variation in the proportion of muscle fiber types (Schiaffino and Reggiani, 2011). Muscle fiber types are defined as the stereotyped covariation of myosin isoforms, sarcoplasmic reticulum (SR) morphology and metabolic enzymes (Schiaffino and Reggiani, 2011). Typical vertebrate twitch muscle fibers are categorized as type I, IIa, IIb or IIx. Type I fibers have slow myosin isoforms, a less-developed SR and oxidative metabolism; type IIb or IIx fibers have faster myosin isoforms, a more developed SR and glycolytic metabolism; and type IIa fibers are intermediate between type I and IIb/x. The forelimb of slow-moving sloths contains many I fibers, whereas fast-moving cheetahs have more type IIb fibers. The stereotyped covariation of myosin isoforms, SR morphology and metabolic enzymes is thought to underpin the speed–endurance trade-off (Garland, 1988; Vanhooydonck et al., 2001; Bonine et al., 2005).

Some muscles and species exhibit a broader range of muscle fiber types than this typical I/IIa/IIb system including tonic fibers, masticatory fibers (Philippi and Sillau, 1994; Hoh, 2002; Schiaffino and Reggiani, 2011; Talbot and Maves, 2016) and hybrid fibers in which more than one myosin isoform is expressed (Larsson and Moss, 1993; Bottinelli and Reggiani, 2000; Korfage et al., 2005; Curry et al., 2012; Medler, 2019; Kohn, 2014; Nguyen et al., 2020). Masticatory fibers are twitch fibers that have rapid calcium sequestration from the SR and contain fast masticatory myosin isoforms (IIm) (Taylor et al., 1973; Hoh, 2002; Rowlerson et al., 1981; Bárány, 1967; Toniolo et al., 2008). Tonic fibers are a categorically different type of muscle fiber than twitch fibers. They have a minimal SR morphology (Hess, 1965; Franzini-Armstrong, 1984), are metabolically similar to slow twitch fibers (Wilkinson and Nemeth, 1989) and can produce sustained contracture (Guttman, 1966; Millman, 1967; Hess, 1970; Cochrane et al., 1972; Bormioli et al., 1979, 1980; Walrond and Reese, 1985; Fisher, 2010). These more specialized fibers and hybrid fibers may expand the function range of muscles and could allow for the circumvention of trade-offs.

**Fig. 1.**
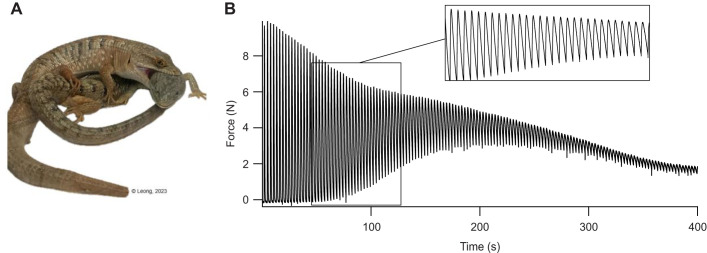
The seasonal mate-holding behavior of the southern alligator lizard (*Elgaria multicarinata*) and the sustained force phenomenon. (A) Image of *E*. *multicarinata* performing mating behavior. (B) Representative fatigue profile from Nguyen et al. (2020) showing zoomed in portion of rising baseline force in black box from 60 to 120 s.

The sustained force produced by the amplexus muscle of various frog species (Eberstein and Sandow, 1961; Rubenstein et al., 1983; Peters, 1994, 2001; Peters and Aulner, 2000; Clark and Peters, 2006; Navas and James, 2007; Ishii and Tsuchiya; 2010; Bowcock et al., 2009) and the jaw muscles of the southern alligator lizard (Nguyen et al., 2020) may be explained by the presence of these less commonly considered fiber types, and the properties of these fibers and the presence of hybrid fibers may allow for the circumvention of trade-offs (Larsson and Moss, 1993; Bottinelli and Reggiani, 2000; Korfage et al., 2005; Curry et al., 2012; Medler, 2019; Kohn, 2014; Nguyen et al., 2020). In sustained force, active muscle force stops returning to baseline between repeated contractions (Shamarina, 1962; Kirby, 1983; Rubenstein et al., 1983; Peters, 1994, 2001; Peters and Aulner, 2000; Clark and Peters, 2006; Navas and James, 2007; Ishii and Tsuchiya, 2010; Nguyen et al., 2020). In the frog forelimb muscles, this production of sustained force is sexually dimorphic and seasonal, observed only in the males during the breeding season, where it likely functions as a form of high endurance when constant rather than cyclical force is required, and enables male frogs to grasp onto the female with their forelimbs for up to 2 weeks (Eberstein and Sandow, 1961; Rubenstein et al., 1983; Peters, 1994, 2001; Peters and Aulner, 2000; Clark and Peters, 2006; Navas and James, 2007; Ishii and Tsuchiya, 2010; Bowcock et al., 2009). Prolonged mating behavior (Pauly, 2018) and sustained force (Fig. 1) (Nguyen et al., 2020) has also been demonstrated in the jaw muscles of *E. multicarinata*; however, its function here is a little less clear as sustained force is exhibited by the jaw muscles of both males and females year-round. Regardless, in addition to high-endurance mate-holding, the jaw muscles are also involved in faster behaviors such as prey capture, and so may need to circumvent the speed–endurance trade-off. The amplexus muscles of male frogs have been shown to contain both fast and tonic fibers (Melichna et al., 1972; Oka et al., 1984), and the jaw muscle of the southern alligator lizard are made up of hybrid fibers containing both tonic and masticatory myosins (Nguyen et al., 2020). Hence, it seems plausible that tonic fibers or myosins produce sustained force whereas faster fibers or myosins allow for more rapid movements (Peters and Aulner, 2000).

In more typical muscles, contractile speed and endurance would simply reflect the balance of fiber types, and a speed–endurance trade-off would likely be visible under strong selection for speed or endurance (Andersen and Henriksson, 1977; Rivero et al., 1993; Bonine et al., 2005; Lacerda de Albuquerque et al., 2015; Scales and Butler, 2016; Castro et al., 2024). However, sustained force is an unusual form of endurance, whereby rather than relying on oxidative metabolism to allow for many repeated contractions, force is instead maintained between contractions. Hence, it may be that a relatively low fraction of tonic myosins is able to sustain some force whereas masticatory myosins may allow for rapid contraction, thus circumventing the speed–endurance trade-off and potentially allowing organisms to fulfil other functions such as the processing of fast prey. To address this question, we examined the rates of isometric force development and relaxation, and force–velocity (FV) properties of the jaw muscles of *E. multicarinata* as metrics of contractile speed (Hill, 1938; Alcazar et al., 2019; Mendoza et al., 2023) and compared these with a more typical locomotor muscle in the thigh, the iliotibialis 2 (IT2). If the jaw muscles of *E. multicarinata* are subject to the speed–endurance trade-off, we expect that they would have much slower velocities than the thigh muscle. However, if sustained force and hybrid fiber types allow these muscles to circumvent this speed–endurance trade-off, we would expect to see more broadly similar contractile speeds in *E. multicarinata* jaw and thigh muscles, as has been observed across species of anoles (Anderson and Roberts, 2020), despite the high endurance conferred by sustained force in the jaw muscle.

## MATERIALS AND METHODS

### Study animals

Adult *E.* *multicarinata* (Blainville 1835) from various counties across the state of California were wild-caught and used in these experiments (*n*=8, jaw; *n*=8, thigh; Specific Use Permit ID: S-203040004-20328-001). Lizards were maintained in the vivarium at the University of California, Riverside, kept in terraria under controlled temperature and light conditions (24±2°C; 12 h:12 h light:dark) with cover objects for hiding, fed calcium-dusted and vitamin-supplemented crickets 3 days per week, and provided with water *ad libitum*. All procedures in the study were approved by the Institutional Animal Care and Use Committees at the University of California, Riverside.

### Muscle preparations

Before beginning experiments, animals were deeply anesthetized with isoflurane (SomnoSuite Low-flow Anesthesia System, Kent Scientific, Torrington, CT, USA), followed by euthanasia using a double-pithing protocol (Gebhart et al., 1992). Then, either the jaw–adductor complex or the thigh (IT2) muscle was isolated and subjected to *in situ* or *in vitro* testing, respectively.

#### Jaw muscle

The jaw–adductor complex was exposed by removing the integument overlying the lateral temporal fenestration on one side of the head. The mandible was cut, freeing the insertion of this muscle complex, and tied with Kevlar. The trigeminal nerve was exposed ventrally by reflecting the overlying jaw–adductor complex (Robison and Tanner, 1962; Haas, 1973). Nerve branches were freed from the musculature using fine tip forceps and the nerve was tied off with 6-0 silk proximally. A hook electrode was place on the nerve to allow for stimulation. Nerve, rather than plate electrode, stimulation was chosen to avoid potential issues with tonic fibers not conducting action potentials (Miledi et al., 1971). The lizard's head and neck were clamped in a customized stereotaxic platform to anchor the origin of the muscle complex, and the distal end of the muscle was connected to the force and length transducer with Kevlar thread (305C-LR Dual Mode Lever System, Aurora Scientific, Aurora, ON, Canada; force range: 10 N; force resolution: 1 mN), allowing for measurements of muscle force, length and velocity (Castro et al., 2022). The exposed muscles were frequently irrigated with Ringer's solution (NaCl 6.545 g, KCl 0.246 g, CaCl_2_ 0.277 g, MgCl_2_ 0.095 g, HEPES 4.766 g, glucose 0.901 g per 1 liter of DI water) during the experiments (Jayasinghe and Launikonis, 2013). All experiments were conducted at ∼24°C. The complex architecture of this muscle, the need for nerve rather than plate stimulation, and preliminary experiments suggesting that fiber bundles extracted from this muscle are relatively fragile and do not survive well *in vitro* necessitated the use of *in situ* muscle preparation in which the whole muscle and nerve could be kept intact and a blood supply maintained. At the end of the *in situ* experiments, the thoracic cavity was exposed to confirm that the heart was still beating and a blood supply to the muscle had been maintained. Amphibian and reptilian hearts can beat for hours after brain death, much longer than mammalian hearts (Leary et al., 2013).

#### Thigh muscle

The IT2 muscle, a knee extensor (Anzai et al., 2015; James et al., 2015), was isolated, the proximal tendon was tied tightly with Kevlar thread and the tendon was cut proximally. The tibia and femur were cut, freeing the distal end of the muscle and a small piece of bone. The distal bone was clamped, and the proximal end of the muscle was connected to the force and length transducer with Kevlar thread (305C-LR Dual Mode Lever System, Aurora Scientific; force range: 10 N; force resolution: 1 mN), allowing for measurements of muscle force, length and velocity (Castro et al., 2022). The muscle was then immersed in a bath containing oxygenated Ringer's solution (as described previously) with platinum plate electrodes on either side of the muscle to allow for stimulation. All experiments were conducted at ∼24°C.

### Determination of contractile properties

#### Muscle stimulation

For the jaw muscle, square wave pulses of 0.1 ms duration and sufficient amplitude to elicit maximum muscle force were delivered (IgorPro 9, WaveMetric, Lake Oswego, OR, USA) to the trigeminal nerve via hook electrodes (CompactDAQ, National Instruments, Austin, TX, USA; Isolated Pules Stimulator Model 2100, A-M Systems, Carlsborg, WA, USA). Single pulses were used for all twitch contractions, whereas 400 ms trains of pulses were delivered at 50 Hz to elicit tetanic contractions. Preliminary experiments indicated that this was the lowest stimulation frequency that reliably resulted in fused contractions and thus maximum force.

For the thigh muscle, square wave pulses of pulse duration 0.1 ms and sufficient amplitude to elicit maximum muscle force delivered (IgorPro 9, WaveMetric) to the muscle via plate electrodes (CompactDAQ, National Instruments, Austin, TX, USA; High-Power, Biphase Stimulator, Aurora Scientific, Aurora, ON, Canada). The use of plate electrodes required the use of a stimulator that could deliver a higher voltage/current than used for nerve stimulation. Single pulses were used for all twitch contractions, whereas 400 ms trains of pulses were delivered at 80 Hz to elicit tetanic contractions. Preliminary experiments indicated that this was the lowest stimulation frequency that reliably resulted in fused contractions and so, maximum force.

#### Muscle isometric contractile properties

Muscle force and length during contractions were logged at 1000 Hz for the jaw muscle and 10,000 Hz for the thigh muscle (IgorPro 9, WaveMetric; CompactDAQ, National Instruments). The need to construct stimulus outputs for the high-power stimulator used for the thigh muscle *in vitro* necessitated this higher sampling frequency. We began each experimental session with a series of twitch contractions at increasing voltages to establish maximal voltage. The lowest stimulus voltage giving peak twitch force was used for all subsequent contractions (1–3 V for jaw and 7–20 V for thigh). Twitch contractions were then performed at varying lengths to establish the muscle length resulting in the maximum force output. This length was defined as optimum length (*L*_0_), and all subsequent contractions were performed at this length. Although twitch and tetanic optimum lengths vary slightly (Askew and Marsh, 1997; Holt and Azizi, 2014), twitch contractions were used in order to preserve the integrity of the muscle while allowing measurements to be made at consistent relative lengths across individuals and muscles. Twitch optimum length is typically slightly longer than tetanic optimum length, hence the use of this length means that the muscle shortens across the plateau of the tetanic force–length relationship during shortening (Askew and Marsh, 1997; Holt and Askew, 2012).

An additional twitch contraction was performed at *L*_0_ to allow for determination of twitch times at a comparable length and point in the experiment across subjects. Isometric tetanic contractions were then performed, first to establish peak force and later to check the viability of the muscle throughout the experiment. Isometric tetanic contractions were performed after every three to four isotonic contractions during the FV protocol, and the maximum force output was compared with the original isometric tetanus contraction (Holt and Azizi, 2014). If force had dropped by ∼30% of the first control isometric tetani, the experiment was terminated. This permitted force drop is greater than is typical (10–20%) (Cairns et al., 2008; Nocella et al., 2011). The jaw muscle appears to be unusually fragile, and it has previously been demonstrated that there is no effect of allowing more fatigue provided the declining maximum performance is accounted for (Bahlman et al., 2020), and so this approach may allow for the study of these more fragile muscles. To account for fatigue, predicted isometric force was calculated for each isotonic contraction assuming a linear decline between control tetanic contractions, and normalized isotonic force was calculated relative to this predicted isometric force.

#### Muscle force–velocity properties

Isotonic tetanic contractions were performed in which the resistive force the muscle experienced was varied (∼0.05–0.8% of maximum isometric force) and the muscle was allowed to shorten. The shortening velocity that could be achieved at these forces was then determined from muscle length recordings.

### Morphological measurements

At the conclusion of the experiments, muscle length (Kynup Digital Caliper; measurement range: 0–150 mm; accuracy: 0.02 mm; resolution: 0.01 mm), body mass (Ohaus Scout SPX421; maximum capacity: 420 g; resolution: 0.1 g) and muscle mass (Mettler Toledo ME 103E; maximum capacity: 120 g; resolution: 0.001 g) were recorded. The muscle was pinned at optimal length on an agar-coated Petri dish and placed under a dissection scope (Leica MZ125 Dissection Stereomicroscope; resolution: 375 line-pairs mm^−1^) to measure muscle fiber length with calipers. Data are presented as means±s.e.m. Once the mass and muscle fiber length were obtained, the physiological cross-sectional area [PCSA=*M*/(ρ*L*_f_), where *M* is muscle mass, ρ is muscle density and *L*_f_ is fiber length] was calculated assuming a density of 1060 kg m^−3^ (Mendez and Keys, 1960). For the jaw experiments, the lizard's thoracic girdle region was exposed to confirm that the heart was still beating to ensure that the jaw muscles were supplied with blood for the duration of the experiment. Lastly, to determine the sex of the animal, a midline laparotomy was performed to reveal the reproductive organs (oviducts and testes).

### Data analysis

Twitch and tetanic rise and relaxation times were recorded from the representative isometric twitch and tetanic contractions performed. Peak force was determined and time series data were used to calculate the time from onset of muscle force production to peak tension, the time from onset of muscle force production to 90% of peak tension, the time from peak tension to 50% relaxation, and the time from peak tension to 90% relaxation (Marsh and Bennett, 1986; Bennett et al., 1989; Askew and Marsh, 1997; Syme et al., 2005; Nguyen et al., 2020; Castro et al., 2022). We calculated the peak isometric tetanic stress (stress=*F*_0_/PCSA) of the muscles (Askew and Marsh, 1997; Zhan et al., 1999; Syme et al., 2005; Holt et al., 2014). Twitch/tetanic force ratios were calculated for jaw and thigh muscles (Celichowski and Grottel, 1993; Askew and Marsh, 1997).

For isotonic FV contractions, velocity (*V*) was normalized to *L*_0_ to yield relative velocity (*L*_0_ s^−1^). Force (*F*) was normalized to peak isometric tetanic force of the muscle (*F*_0_) to yield relative isometric force *F*/*F*_0_ (Castro et al., 2022). Fatigue was determined as the decline in force between subsequent isometric control contractions, and only data points before the muscle reached 30% fatigue were included (Bahlman et al., 2020). The fragility of this muscle meant that a sufficient number of points to construct a complete FV curve could not be obtained on all individuals. Hence, the normalized FV data points from all individuals were collated (Holt et al., 2014) and fit with a Marsh–Bennett hyperbolic-linear equation (Marsh and Bennett, 1986) (Eqn 1; constants *B* and *C* have dimensions of velocity, and constant *A* is dimensionless), and predicted maximum unloaded shortening velocity (*V*_max_) was obtained (Marsh and Bennett, 1986; Bennett et al., 1989; Askew and Marsh, 1997; Zhan et al., 1999; Syme et al., 2005; Holt et al., 2014; Alcazar et al., 2019; Javidi et al., 2020; Castro et al., 2022):
(1)

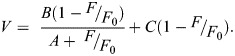


Power curves were calculated from the force and velocity points from the FV data fit with the Marsh–Bennett equation [Power (W)=*FV*, where *F* is force (N) and *V* is velocity (m s^−1^)]. Peak power was collected for each of the curves. The power ratio defined in Marsh and Bennett (1986) was used to determine curvature of the FV relationships [*V*=*W*_max_/(*V*_max_*F*_0_), where *W*_max_ is maximum power output). Faster muscles are associated with higher power ratios and greater curvature and vice versa for slower muscles (Marsh and Bennett, 1986; Mendoza et al., 2023).

### Statistical analysis

All analyses were performed in RStudio (Integrated Development Environment for R Posit Software, PBC, Boston, MA, USA). The significance level used to test for differences between the jaw and thigh muscle contractile times was 0.05.

Data were tested for normality using the Shapiro–Wilk test and for equal variances using *F*-tests. Twitch time from onset of muscle force production to 90% of peak tension and time from peak tension to 90% relaxation, and tetanic time from onset of muscle force production to 90% of peak tension were normally distributed, and so a parametric, unpaired two-sample *t*-test was used to compare these variables between the jaw and thigh muscles. Twitch time from onset of muscle force production to peak tension and time from peak tension to 50% relaxation, and tetanic time from onset of muscle force production to peak tension, time from peak tension to 50% relaxation, and time from peak tension to 90% relaxation data, in contrast, were non-normally distributed. Box–Cox, logarithmic and square root transformations were attempted to normalize these data. However, the transformed data remained non-normally distributed and the nonparametric Mann–Whitney *U*-test was used to compare these variables between the jaw and thigh muscles.

The FV data were modeled with a mixed effects model using a gamma distriution and a log-link function with a 95% confidence interval (see Fig. S1). Our study contains multiple measurements per individual thus a mixed model analysis was necessary. Relative velocity was the dependent variable as it was measured at different set forces, and the independent variables were relative force and muscle type (fixed effects) and individual (random effect).

## RESULTS

### Body size and muscle dimensions

The average body mass for individuals used for the jaw and thigh measurements, and muscle fiber length, muscle mass, PCSA for the jaw and thigh muscles used in the experiments are summarized in Table 1.

**Table 1.** Mean±s.e.m. of body metrics and muscle dimensions in *Elgaria multicarinata*

	Jaw (*n*=8)	Thigh (*n*=8)
Body mass (g)	39.7±3.7	49.7±3.4
Muscle fiber length (mm)	11.50±0.26	6.17±0.55
Muscle mass (g)	0.443±0.043	0.078±0.014
Physiological cross-sectional area (cm^2^)	0.37±0.04	0.13±0.03

### Isometric properties

The average stress value for the tetanic contractions for the jaw muscle was 7.4±1.3 N m^−2^ (*n*=8) and for the thigh muscle was 16.2±3.7 N m^−2^ (*n*=8), and stress was significantly different between the jaw and the thigh muscle (*P*=0.04163, *t*=−2.2426, d.f.=14). The twitch/tetanus force ratio was lower for the jaw muscle (0.205±0.055) than for the thigh muscle (0.219±0.023), but there was no significant difference (*P*=0.818, *t*=−0.234, d.f.=14).

**Fig. 2.**
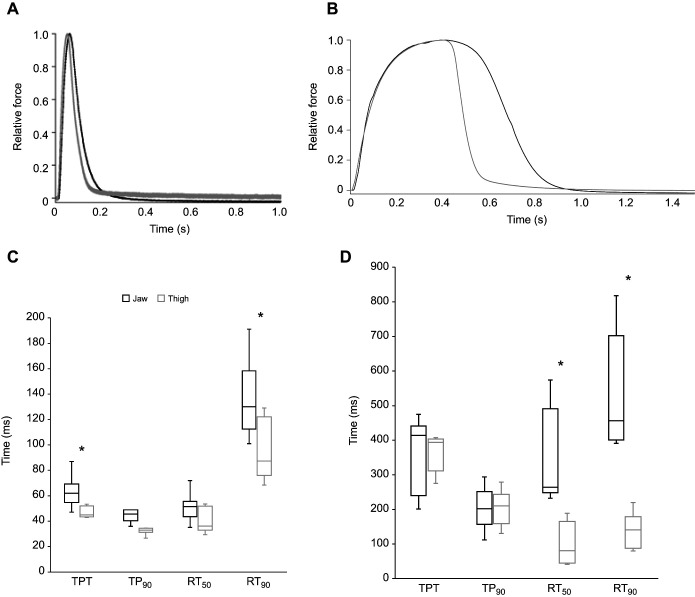
**Twitch and tetanic traces and contraction times of the southern alligator lizard (*Elgaria multicarinata*) jaw and thigh muscles.** Representative twitch (A) and tetanic (B) contraction traces and corresponding summary boxplots of twitch (C) and tetanic (D) contraction times of the jaw (black; *n*=8) and thigh muscle (grey; *n*=8) of *E. multicarinata*. TPT, time to peak tension; TP_90_, time to 90% of peak tension; RT_50_, time to 50% relaxation, measured from peak to 50% relaxation; RT_90_, time to 90% relaxation, measured from peak to 90% relaxation. *Statistically significant difference (*P*<0.05). There was a significant effect of muscle on (C) twitch TPT (*P*=0.01359, *W*=56) and RT_90_ (*P*=0.009012, *t*=3.0293, d.f.=14), and (D) tetanic RT_50_ (*P*=0.0009391, *W*=64) and RT_90_ (*P*=0.000931, *W*=64).

Representative normalized isometric twitch and tetanic contractions time courses are shown (Fig. 2A,B). Twitch contraction times for the jaw muscle show that time from onset of muscle force production to peak tension was 63±4 ms, time from onset of muscle force production to 90% of peak tension was 46±3 ms, time from peak tension to 50% relaxation was 51±4 ms, and the time from peak tension to 90% relaxation was 136±11 ms (*n*=8; Fig. 2C). For the thigh muscle, twitch time from onset of muscle force production to peak tension was 48.5±3.0 ms, twitch time from onset of muscle force production to 90% of peak tension was 33.7±2.0 ms, twitch time from peak tension to 50% relaxation was 40.7±3.5 ms, and twitch time from peak tension to 90% relaxation was 95.7±8.2 ms (*n*=8; Fig. 2C). Twitch time from onset of muscle force production to peak tension was significantly longer in the jaw than in the thigh muscle (*P*=0.01359, *W*=56), but twitch time from onset of muscle force production to 90% of peak tension was not significantly different between the two muscles (*P*=0.2589, *t*=1.1767, d.f.=14). Twitch time from peak tension to 90% relaxation was significantly longer in the jaw than in the thigh (*P*=0.009, *t*=3.0293, d.f.=14), but twitch time from peak tension to 50% relaxation was not significantly different between the two muscles (*P*=0.1036, *W*=48).

Tetanic contraction times for the jaw show that time from onset of muscle force production to peak tension was 364±38 ms, time from onset of muscle force production to 90% of peak tension was 204±21 ms, time from peak tension to 50% relaxation was 348±49 ms, and time from peak tension to 90% relaxation was 537±59 ms (*n*=8; Fig. 2D). For the thigh muscle, tetanic time from onset of muscle force production to peak tension was 364.2±128.8 ms, tetanic time from onset of muscle force production to 90% of peak tension was 206.7±17.6 ms, tetanic time from peak tension to 50% relaxation was 98.2±34.7 ms, and tetanic the time from peak tension to 90% relaxation was 139.6±19.1 ms (*n*=8; Fig. 2D). The tetanic time from onset of muscle force production to peak tension (*P*=0.3184, *W*=42) and tetanic time from onset of muscle force production to 90% of peak tension (*P*=0.9116, *t*=−0.11302, d.f.=14) were not significantly different between the jaw and thigh muscles. Tetanic time from peak tension to 50% relaxation (*P*=0.0009391, *W*=64) and tetanic time from peak tension to 90% relaxation (*P*=0.000931, *W*=64) were significantly longer in the jaw than in the thigh muscle.

### Force–velocity properties

**Fig. 3.**
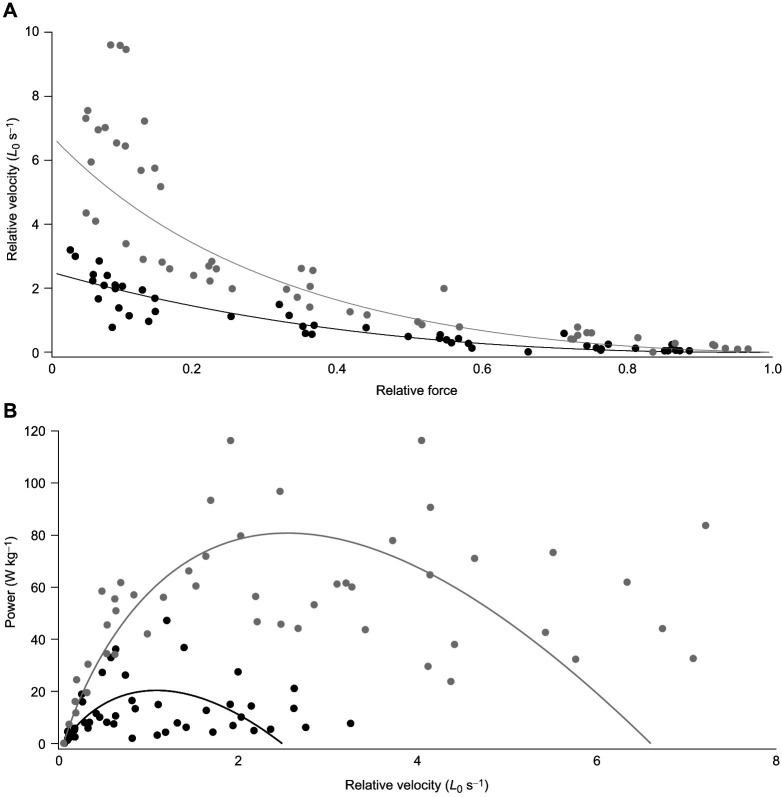
**Relative force–velocity and relative velocity--power traces of the southern alligator lizard (*****Elgaria multicarinata*****) jaw and thigh data.** (A) Force–velocity data points normalized to muscle length fitted with a Marsh–Bennett curve fit for the jaw (*n*=8; black markers; *V*_max_=2.44±0.24*L*_0_ s^−1^) and thigh (*n*=8; grey markers; *V*_max_=6.95±1.03*L*_0_ s^−1^) muscle of *E. multicarinata*. (B) Power–velocity curves and fits constructed using raw force and velocity points and Marsh–Bennett curve fit for jaw (peak power=20.4 W kg^−1^; power ratio=0.12; black) and thigh (peak power=80.8 W kg^−1^; peak ratio=0.11; grey).

There was a significant effect of muscle type (i.e. jaw versus thigh) on velocity (*P*=9.661343e-07; Fig. 3; Fig. S1), with the jaw muscle (*n*=8; *V*_max_=2.44±0.24*L*_0_ s^−1^, peak power=20.4 W kg^−1^, power ratio=0.12) being slower than the thigh (*n*=8; *V*_max_=6.95±1.03*L*_0_ s^−1^, peak power=80.8 W kg^−1^, power ratio=0.11). However, although there was an effect of muscle type on shortening velocity, it does not appear as though there was an effect on the shape of the force–velocity relationship as there is no significant interactive effect of force and muscle type on velocity (*P*=3.683, Fig. 3; Fig. S1). This is reflected in the very similar power ratios for the two muscle types.

## DISCUSSION

This study addresses the question of whether the sustained force developed by the ‘specialized’ jaw muscle of *E. multicarinata*, potentially to facilitate the long-lasting mate-holding behavior, represents a means by which the speed–endurance trade-off commonly thought to occur in skeletal muscle (Komi, 1984; Esbjörnsson et al., 1993; Wilson et al., 2002; Castro et al., 2022; Garland et al., 2022) can be circumvented. We determined the contractile speed of these jaw muscles, both in terms of their rate of force development and relaxation and their shortening velocity, then compared these properties with a more typical thigh muscle used in locomotion (James et al., 2015). If these muscles are subject to the typical speed–endurance trade-off, the jaw muscles would exhibit slow contractile properties compared with the thigh muscle. Twitch time to peak twitch tension, twitch time to 90% relaxation, tetanic time to 50% relaxation, and tetanic time to 90% relaxation were significantly slower in the jaw muscle than in the thigh (Fig. 2). However, tetanic force rise times were not significantly different between these muscles and in general, the two muscles appear to be less different in force rise than force relaxation times (Fig. 2). The jaw muscle also had a significantly lower maximum relative shortening velocity (*V*_max_=2.44±0.24*L*_0_ s^−1^) and peak power (peak power=20.4 W kg^−1^) (Fig. 3). However, there did not appear to be any interactive effect of force and muscle type (*P*=3.683) and the power ratios, a metric of the shape of the force–velocity relationship (Marsh and Bennett, 1986), were very similar in the two muscles being 0.12 in the jaw and 0.11 in the thigh. This is slightly surprising as slower muscles are often though to exhibit a greater degree of curvature in their force–velocity relationship (Schiaffino and Reggiani, 2011). However, the factors determining this shape in whole muscles and *in situ* preparations, as opposed to single fibers, are poorly understood and likely to be multifactorial (Holt et al., 2014; Alcazar et al., 2019).

Our data suggest that the jaw muscle of *E. multicarinata* is slow compared with the thigh muscle. However, a range of contractile velocities has been observed across the phylogeny and with ecology (Mendoza et al., 2023). Here, we compare the jaw muscle of *E. multicarinata* with the classic model of fast and slow muscles, mouse (*Mus musculus*) soleus and extensor digitorum longus (EDL), as well as representative literature for a comparison between a feeding muscle and locomotor in *Anolis* (Anderson and Roberts, 2020), and with other ‘specialized’ slow muscles used in mating behavior and known to exhibit sustained force, and the most extreme slow muscles found in the literature. However, this comparison is complicated by the effects of temperature, with experimental temperature varying widely and having a major effect on contraction speed. To account for this, we give both values as reported in the literature and corrected to match our experimental temperature of 24°C assuming a *Q*_10_ of 2 (Bennett, 1985; Rall and Woledge, 1990; Anderson and Deban, 2010). Any comparisons discussed throughout will be between the data presented here and the values converted to 24°C.

The twitch rise time of the jaw muscle (TPT=63±4 ms at 24°C) is at least 3 times slower than the fast mammalian *M. musculus* EDL muscle (TPT=7.3±0.2 ms at 37°C; TPT≈18.42 ms at 24°C) and at least 1.5 times slower than the slow mammalian *M. musculus* soleus muscle (TPT=16.2±0.4 ms at 37°C, TPT≈40.8 ms at 24°C). Twitch half relaxation time for the *E. multicarinata* jaw muscle (RT_50_=51±4 ms at 24°C) is more than 2 times slower than that of the *M. musculus* EDL (RT_50_=9.1±0.4 ms at 37°C; RT_50_≈22.9 ms at 24°C) but slightly faster than that of the *M. musculus* soleus (RT_50_=23.0±1.0 ms at 37°C; RT_50_≈58 ms at 24°C). The tetanic half relaxation time for the jaw muscle of *E. multicarinata* (RT_50_=348±49 ms at 24°C) was at least 11 times slower than that of the mouse EDL muscle (RT_50_=12.5±4 ms at 37°C; RT_50_≈31.5 ms at 24°C) and at least 3 times slower than that of the *M. musculus* soleus (RT_50_=41.6±2.3 ms at 37°C; RT_50_≈104.9 ms at 24°C) (Askew and Marsh, 1997). The jaw *V*_max_ value of *E. multicarinata* (*V*_max_=2.44±0.24*L*_0_ s^−1^ at 24°C; Fig. 3) was at least 2 times lower than that of the mouse EDL muscle (*V*_max_=14.1±0.8*L*_0_ s^−1^ at 37°C; *V*_max_≈5.6*L*_0_ s^−1^ at 24°C) and the same as that of the *M. musculus* soleus muscle (*V*_max_ of 6.0±0.3*L*_0_ s^−1^ at 37°C; *V*_max_≈2.4*L*_0_ s^−1^ at 24°C) (Askew and Marsh, 1997). If compared with *Anolis* species, the jaw muscle of *E. multicarinata* is slower than the jaw and thigh muscles measured at 28.2–33.4°C depending on the species (TPT=19.8±0.7–55.6±1.5 ms; *V*_max_=6.0±0.5–14.2±1.0*L*_0_ s^−1^) (Anderson and Roberts, 2020).

The ‘specialized’ slow jaw muscle of *E. multicarinata* is slow in comparison with the locomotor muscles used as representative comparisons in the previous paragraphs, with the exception of some metrics of speed in comparison with the slow soleus. If compared with more specialized slow muscles, it does not appear to be exceptionally slow. For example, when comparing with the iliofibularis (IF) hindlimb muscle of the slow-moving chameleon (*Chamaeleo senegalensis*) (TPT=122±35 ms at 23°C; TPT≈113 ms at 24°C), the twitch rise times of *E. multicarinata*'s jaw muscles (TPT=63±4 ms at 24°C; V_max_=2.44±0.24*L*_0_ s^−1^ at 24°C) are almost two times faster, but the *V*_max_ values are similar (*V*_max_ of 2.5±1.1*L*_0_ s^−1^ at 23°C; *V*_max_≈2.68*L*_0_ s^−1^ at 24°C) (Abu-Ghalyun et al., 1988). The twitch rise time of the *E. multicarinata*'s jaw muscles (TPT=63±4 ms at 24°C) is at least 12 times faster, and its *V*_max_ is at least two times higher, than the slowest muscle measured, that of the tortoise (*Testudo graeca* or *T. hermanni*) rectus femoris (RF) hindlimb muscle (TPT=4000 ms at 0°C; TPT≈757 ms at 24°C; *V*_max_=0.23±0.03*L*_0_ s^−1^ at 0°C; *V*_max_≈1.2*L*_0_ s^−1^ at 24°C) (Woledge, 1968).

This slow phenotype of the jaw muscle of *E. multicarinata* is comparable to other muscles that are used in mate-holding behavior and exhibit sustained force. The flexor carpi radialis muscle (FCRM) of the frog (*Rana temporaria*), which is used in amplexus (Thibert and Nicolet, 1975), also exhibits the sustained force observed in *E. multicarinata*'s jaw muscle (Nguyen et al., 2020). The FCRM twitch rise time (TPT=44.2±1.40 ms at 20°C; TPT≈33.4 ms at 24°C) is about two times faster than *E. multicarinata*'s jaw muscle (TPT=63±4 ms at 24°C) and the twitch half relaxation time is a fraction faster (RT_50_=60.6±3.4 ms; RT_50_≈45.7 ms at 24°C) than *E. multicarinata*'s jaw muscle (RT_50_=51±4 ms at 24°C). Both the forelimb muscles of frogs and the jaw muscles of *E. multicarinata* contain both tonic fibers and twitch fibers (Eberstein and Sandow, 1961; Thibert and Nicolet, 1975; Kirby, 1983, Rubenstein et al., 1983; Peters and Aulner, 2000; Nguyen and Stephenson, 2002; Navas and James, 2007; Ishii and Tsuchiya, 2010; Nguyen et al., 2020), with the jaw muscles of *E. multicarinata* (Nguyen et al., 2020) and various frog limb muscles (Thibert and Nicolet, 1975; Nguyen and Stephenson, 2002) having been demonstrated to have ‘hybrid’ fiber types containing both slow tonic and fast masticatory myosin heavy chain isoforms. This combination of slow tonic and fast masticatory myosin heavy chain isoforms likely results in the slow, but not exceptionally slow, contractile properties observed here. The relatively fast rise times compared with very slow relaxation times seen in the jaw muscle of *E. multicarinata* (Fig. 2) might be interpreted as force initially rising relatively rapidly owing to the rapid force generation by masticatory myosin and the slower force generation of tonic myosin that then remain active and sustain force (Fig. 2A,B) (Thibert and Nicolet, 1975; Kirby, 1983).

Here, we showed that the jaw muscle of *E. multicarinata*, which exhibits unusual endurance in the form of sustained force, has slow contractile properties relative to the more typical thigh muscle of this species and other common locomotor muscles. It is important to note that there is a general lack of published data on the jaw musculature, and that the different developmental origins of the jaw and locomotor muscles raise questions about whether observed differences are developmental, adaptive or due to physiological constraints to adaptation (English, 1985; Tokita and Scheider, 2009; Woronowicz and Schneider, 2019; Granatosky and Ross, 2020). Hence, the jaw musculature in general is an area that would benefit from further study. However, when compared with the similarity in the contractile speed of the jaw and the thigh across several species of anoles (Anderson and Roberts, 2020), the differences observed between the jaw and thigh here suggest that the relatively slow contractile properties of the jaw muscle we observe are due to a speed–endurance trade-off.

Despite the apparent inability to entirely circumvent the speed–endurance trade-off, it is possible that the combination of slow tonic and fast masticatory myosin may enable the jaw muscles of *E. multicarinata*, and the forelimb muscles of frogs used in amplexus, to meet the conflicting physical demands placed on these muscles. In general, there seems to be less of an effect on activation compared with relaxation, especially in tetanic contractions. This relatively rapid rise in force, presumably owing to the activation of masticatory myosin, may allow for them to quickly grasp onto their targets, whether it be prey or their mates, whereas the prolonged force production, presumably owing to tonic myosins, may give rise to sustained force and the ability to perform these long-lasting mating holding behaviors.

## Supplementary Material

10.1242/jexbio.247979_sup1 Supplementary information
