# Supplementary material for: Does the unusual phenomenon of sustained force circumvent the speed–endurance trade-off in the jaw muscle of the southern alligator lizard (Elgaria multicarinata)?
Source: J Exp Biol. 2025 Jan 27;228(2):JEB247979. doi: 10.1242/jeb.247979 (PMC11832124; doi:10.1242/jeb.247979)
Supplement: Supplementary information [file jexbio-228-247979-s1.pdf]

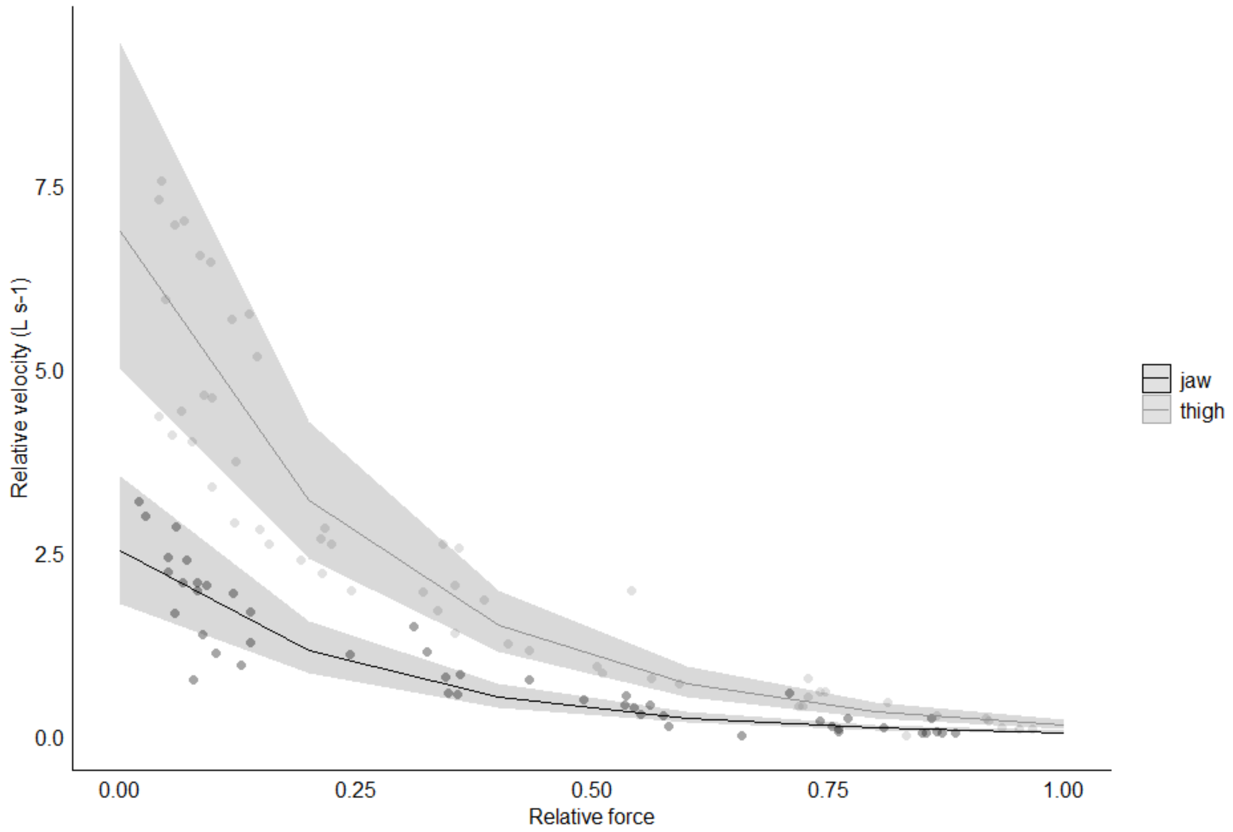

Fig. S1. Predicted values of relative velocities using a mixed-effects model with Gamma distribution and log-link function based off 95% confidence interval of the force-velocity data between jaw (n=8) and thigh (n=8) of the *E. multicarinata*.

Journal of Experimental Biology • Supplementary information
